# A porcine *ex vivo* model of pigmentary glaucoma

**DOI:** 10.1038/s41598-018-23861-x

**Published:** 2018-04-03

**Authors:** Yalong Dang, Susannah Waxman, Chao Wang, Ralitsa T. Loewen, Ming Sun, Nils A. Loewen

**Affiliations:** 1 Department of Ophthalmology, School of Medicine, University of Pittsburgh, Pittsburgh, United States of America; 2 Department of Ophthalmology, Xiangya Hospital, Central South University, Changsha, China; 3 Department of Cell Biology, School of Medicine, University of Pittsburgh, Pittsburgh, United States of America

## Abstract

Pigment dispersion can lead to pigmentary glaucoma, a poorly understood condition of younger myopic eyes with fluctuating high intraocular pressure. It has been difficult to investigate its pathogenesis without a model similar to human eyes in size and behavior. Here we present a porcine *ex vivo* model that recreates several features of pigmentary glaucoma, including intraocular hypertension, accumulation of pigment in the trabecular meshwork, and declining phagocytosis. We found that trabecular meshwork cells regulate outflow, form actin stress fibers, and have a decreased phagocytic activity. Gene expression microarrays and a pathway analysis of TM monolayers as well as *ex vivo* anterior segment perfusion cultures indicated that RhoA plays a central role in regulating the cytoskeleton, motility, and phagocytosis in the trabecular meshwork, providing new insights and targets to investigate in pigmentary glaucoma.

## Introduction

**Figure 1:**
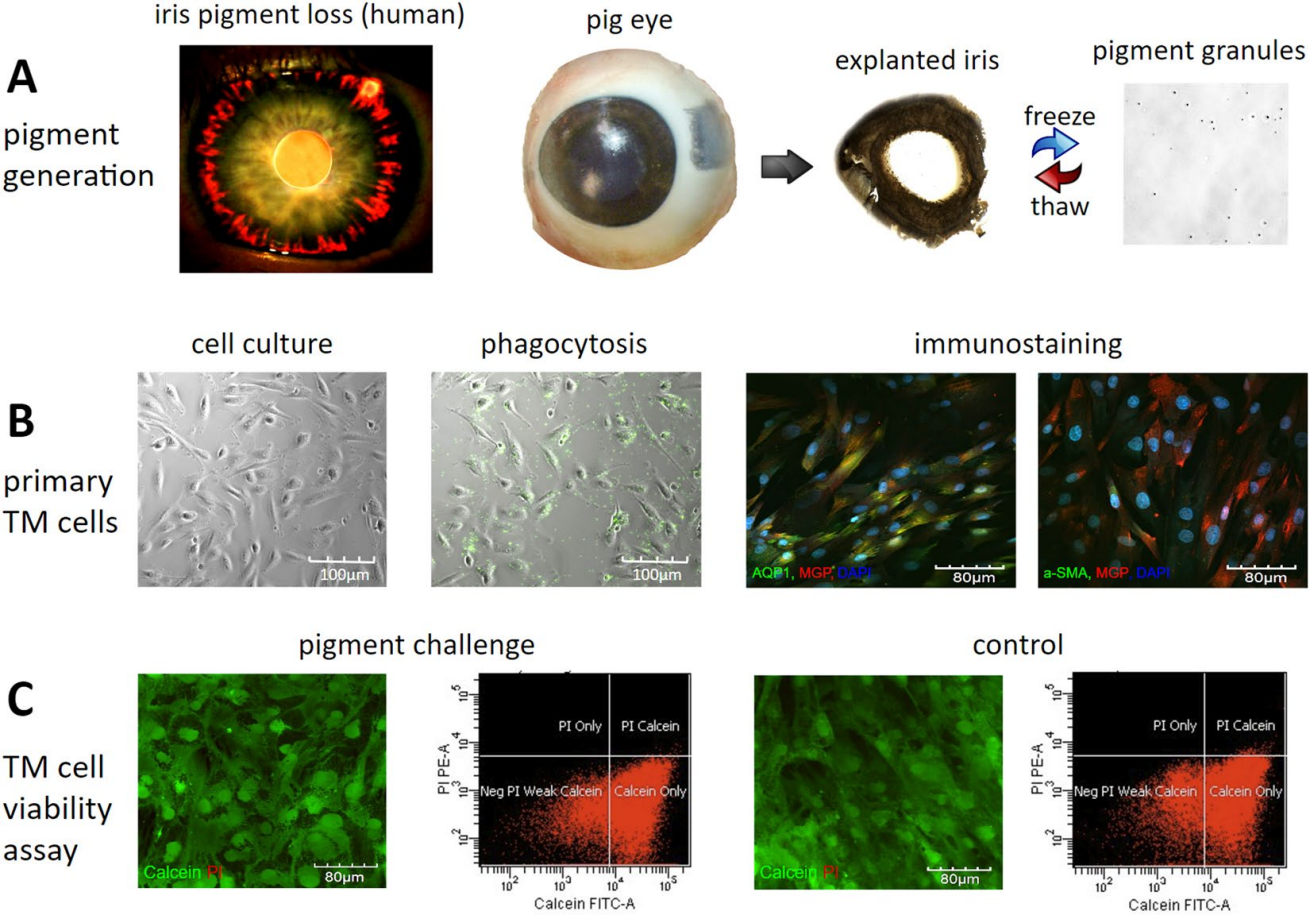
Pigment generation and *in vitro* exposure to pigment dispersion. In the human eye with pigment dispersion, pigment and stroma are lost in the mid-periphery of the iris (transillumination, (**A**) left). Similar pigment granules can be generated by exposing an explanted pig iris to freeze-thaw cycles (**A**, middle and right). The granules had a mean size of 1.03 ± 0.11 microns (**A**, right, single hemocytometer grid shown). Isolated primary trabecular meshwork cells from pig eyes (**B**, left to right) displayed the characteristic morphology, phagocytic activity (fluorescent microspheres), and immunostaining pattern with trabecular meshwork-specific markers, i.e., matrix Gla protein, AQP1, and alpha-SMA (**B**, right). Exposure to pigment did not change the percentage of viable cells or propidium iodide-positive, dead, or apoptotic cells (**C**).

Pigmentary glaucoma (PG) is a secondary open-angle glaucoma in myopic eyes that affects people in their 30 s to 40s^1^. Patients with PG often experience fluctuating intraocular pressures (IOP) that can be high and more resistant than primary open-angle glaucoma to non-surgical treatment^1,2^. In addition to a baseline dispersion of pigment, physical activity^3,4^ or eye movements can trigger pigment showers in some patients, but often without symptoms, which makes this condition particularly vexing. First described by Sugar and Barbour in 1949^5^, the clinical hallmarks of pigment release are readily apparent and include transillumination of the mid-peripheral iris (Fig. 1), deposition of pigment on the corneal endothelium (Krukenberg’s spindle), and in the trabecular meshwork (TM)^6^. The pathogenesis of pigment dispersion remains poorly understood; however, it seems to be caused by mutations or variants of more than one gene. Although a susceptibility locus was mapped to chromosome 7q35–q36, a specific candidate gene has yet to be identified^7^.

The amount of pigment granules in the aqueous humor is correlated with IOP^8^, but the amount observed^9^ is insufficient for a simple physical outflow obstruction as a primary mechanism. Models of pigment dispersion include the DBA/2J^10^ mouse that experiences ocular hypertension following synechial angle closure, iris atrophy, and pigment dispersion^10^. In contrast, Col18a1(−/−) mice^11^ have a collagen XVIII/endostatin deficiency that leads to pigment dispersion via an unknown mechanism and lacks ocular hypertension. Mouse eyes have a limited number of TM layers and are approximately 455 times smaller than human and porcine eyes^12^, making *ex vivo* cultures more challenging^13^. Monkeys can develop an elevated IOP in response to repeated intracameral pigment injections^14^, but concentrated bolus applications do not reflect the chronic pigment release in PG well. Bolus injections of pigment in normal rodent eyes would be difficult to perform because of the small anterior chamber volume of only a few microliters.

In our previous work with pig eyes and the study presented here, we took advantage of the high tissue quality that is the result of only two hours from enucleation to culture, the consistency within a litter, and an outflow tract anatomy that matches several features in humans^15–18^. Notable differences are a thicker TM, Schlemm’s canal-like segments instead of a mostly single lumen (angular aqueous plexus)^19^, and, in contrast to almost all other domestic animals and pets^20^, a paucity of naturally developing glaucoma or medically-induced ocular hypertension. We recently established gene transfer^17,21^, modeled segmental aqueous outflow^16,22,23^, and created a microincisional angle surgery system^24–26^ in a pig eye model.

We hypothesized that *ex vivo* perfused pig eyes would experience reduced outflow in response to continuous exposure to pigment at a concentration far lower (10,000-fold) than that used in previous bolus experiments^14^. The aim of this study was to develop a standardized and accessible PG model that allows studying the function of the TM and examine signal pathway changes to identify new treatment targets.

## Results

We developed a porcine eye model of PG that replicates clinical features including pigment granules in the aqueous humor^8^ and inside of TM cells^9,27,28^.

### Unchanged viability of primary TM cells

The freeze-thaw method produced pigment granules measuring 1.03 ± 0.11 microns in diameter similar to the pigment in human PG. Stocks could be kept at a concentration of 4.3 × 10^9^ particles/ml without clumping **(**Fig. 1A**)**. Primary TM cells obtained from freshly prepared TM tissue had characteristic flat, elongated trabecular shapes^29^ that became more spindle-tipped with increasing confluence and were positive for TM-specific markers, including the matrix Gla protein (MGP)^30–33^, aquaporin 1 (AQP1)^32,34^, and alpha-smooth muscle actin (SMA)^35–37^ **(**Fig. 1B). Myocilin (MYOC) transcription was increased by 40% after exposure to dexamethasone (500 nM) for 7 days.

We tested for any cytotoxicity of the pigment granules by calcein acetoxymethyl (AM) and propidium iodide (PI) co-labeling followed by flow cytometry. The non-fluorescent AM derivative of calcein is transported through the cell membrane into a live cell, whereas PI cannot cross the membrane of a live cell, making it useful to differentiate necrotic, apoptotic, and healthy cells^38^. Viable TM cells can convert non-fluorescent calcein AM to green fluorescent calcein with an intracellular esterase, but do not allow PI to enter or to bind nucleic acids^39^. Pigment granules at a concentration of 1.67 × 10^7^ particles/ml did not increase the percentage of PI-labeled apoptotic or dead cells (0.00 ± 0.00% in the pigment group vs 0.27 ± 0.07% in the normal control, P > 0.05). Similarly, the percentage of calcein-labeled viable cells was not decreased (84.90 ± 3.87% in the pigment group vs 84.57 ± 3.00% in the normal control group, p > 0.05; Fig. 1C).

### Reduction of outflow facility

**Figure 2:**
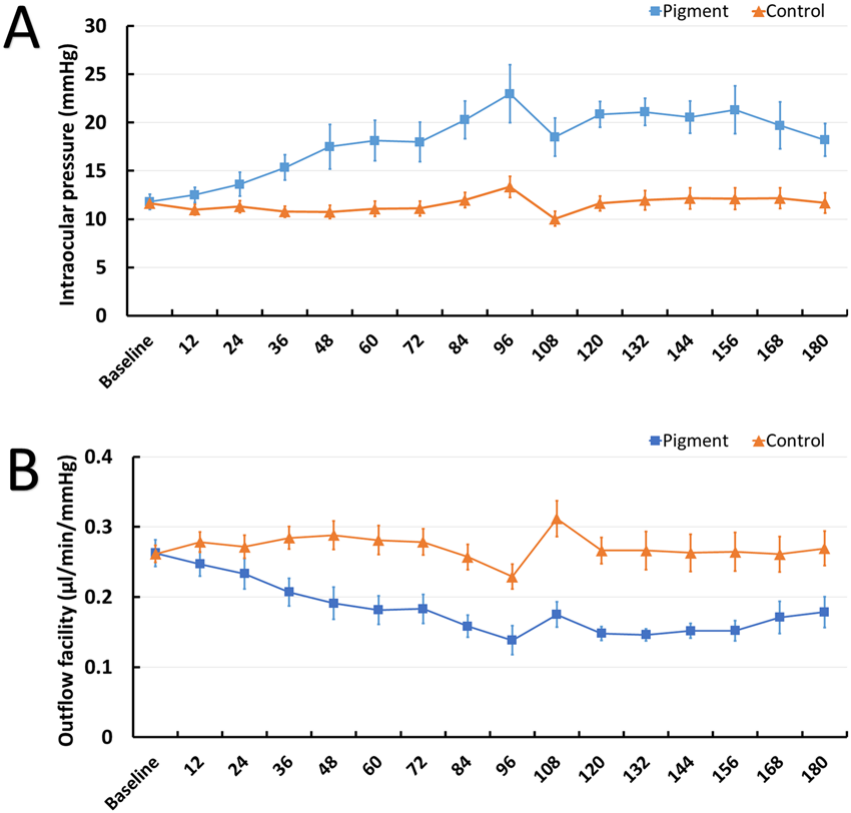
IOP elevation and reduction in outflow facility following pigment dispersion. The IOP of perfused anterior segments started to increase after 48 h (p = 0.026) and persisted for the remainder of the study (all p < 0.05). IOP fluctuation in the pigment group was significantly greater than in the paired controls (p < 0.001). A medium change occurred after the 96-h time point, causing the parallel dip in IOP **(A)**. Outflow facility is inversely correlated with IOP when episcleral venous pressure is zero. Pigment perfusion significantly reduced the outflow facility from the baseline of 0.26 ± 0.02 μl/min/mmHg to 0.21 ± 0.02 μl/min/mmHg at the 36 hours (*P* = 0.01), then throughout the study (all *P* < 0.05, compared to the baseline) **(B)**. IOP, intraocular pressure.

Eight left-right matched porcine anterior segment pairs were randomly assigned to the pigment group or the control group. Similar baseline IOP values were established at 72 hours (11.80 ± 0.78 mmHg in the pigment group and 11.64 ± 0.51 mmHg in the control group, p = 0.872), followed at 180 hours by continued pigment dispersion with a pigment debris granule concentration of 1.67 × 10^7^ particles/ml or a sham treatment with normal tissue culture medium. There was no significant change in IOP in the control group during the experiment (all p > 0.05 vs baseline). In contrast, pigment dispersion resulted in an IOP elevation that became significant at 48 hours, reached a peak of 23.0 ± 6.0 mmHg at 96 hours, and persisted at a level 75% above the baseline of 11.5 ± 0.8 mmHg (all p < 0.05 vs group average at baseline). Eyes with pigment also showed a significantly greater fluctuation in IOP than controls (1.78 ± 0.62 vs 0.83 ± 0.22, p < 0.001), indicating that individual eyes showed variable IOP responses when exposed to the pigment **(**Fig. 2A**)**.

Since the episcleral venous pressure is presumed to be close to zero in this anterior segment perfusion model, the outflow facility was inversely correlated to IOP according to the classic Goldmann equation^40^. The baseline outflow facilities between the two groups were comparable (0.26 ± 0.01 μl/min/mmHg in the control versus 0.26 ± 0.02 μl/min/mmHg in the pigment group, P = 0.95). Pigment perfusion significantly reduced the outflow to 0.21 ± 0.02 μl/min/mmHg after 36 hours (P = 0.01) and persisted (all P < 0.05, compared to the baseline), while the outflow facility of the control group remained unchanged **(**Fig. 2B**)**.

### Lysosome and phagosome activation

**Figure 3:**
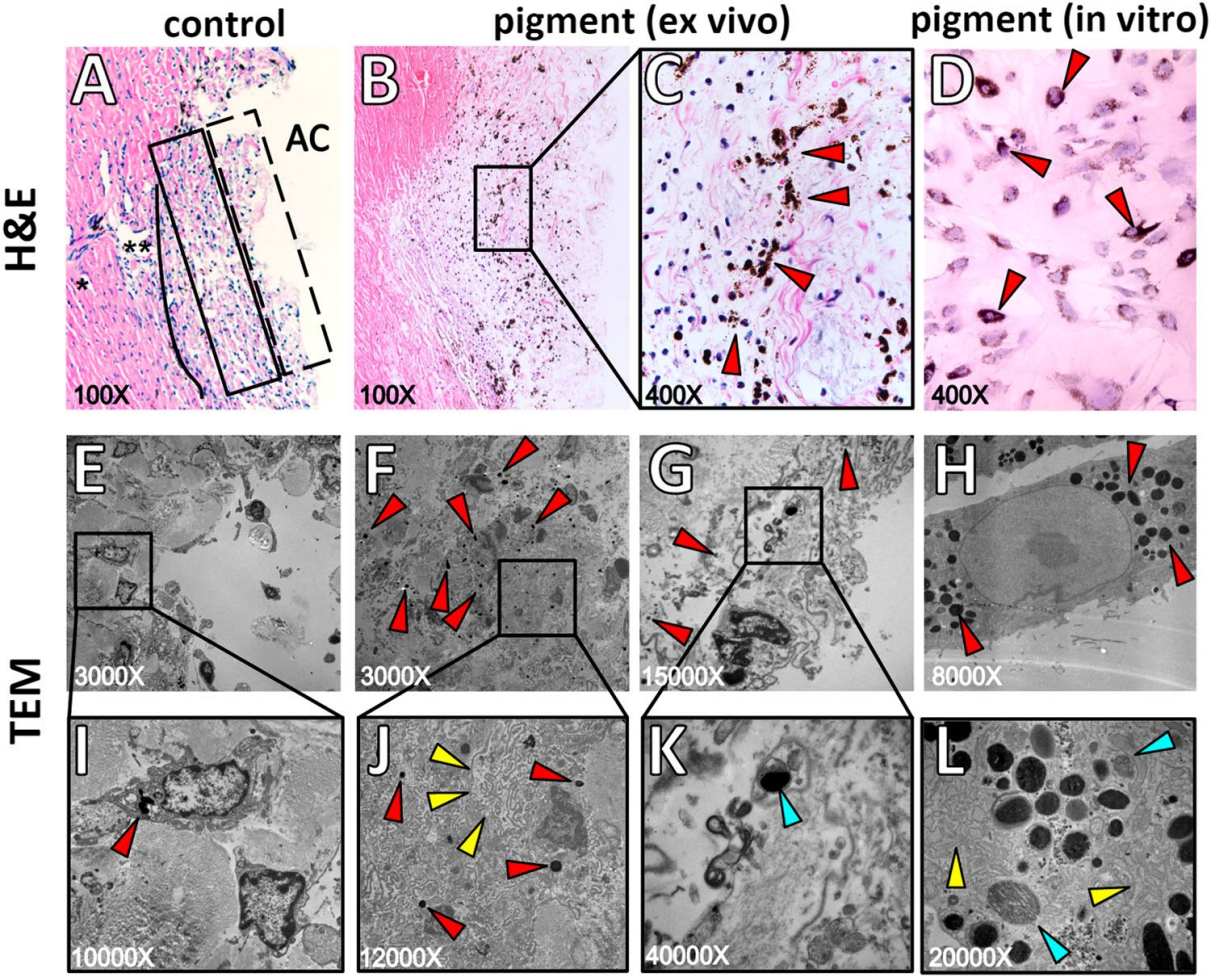
Ultrastructure and histology of the trabecular meshwork. The TM consisted of three characteristic layers: the uveal meshwork (box with dashed line, **A**), the corneoscleral meshwork (box with solid line, **A**), and the juxtacanalicular meshwork (solid line, **A**), adjacent to the inner wall of Schlemm’s canal (**A**, black asterisks). The outer layers were phagocytically active. Pigment granules were located within cells and around the nucleus in the *ex vivo* model (**B,C**, red arrows) and *in vitro* (**D**, red arrows). Transmission electron microscopy showed occasional pigment in the normal TM (**E,I**, red arrows), but a larger number in the inner TM layers (Fig. 3F,J, red arrows), the outer TM layers (**G,K**, red arrows), and in primary TM cells after pigment treatment (**H**). Pigment hydrolysis in different phagolysosome stages (**K**,**L**, blue arrows) and endoplasmic reticulum (**J,L**, yellow arrows) were also seen *in vitro* and *ex vivo*. TM, trabecular meshwork.

The anterior segments of the control eyes had a normal TM consisting of the uveal meshwork, corneoscleral meshwork, and juxtacanalicular region immediately adjacent to the inner wall of Schlemm’s canal-like segments of the angular aqueous plexus (Fig. 3A). Intracellular pigment was rarely seen (Fig. 3I, red arrowhead). In contrast, eyes with pigment dispersion contained many TM cells with intracytoplasmic pigment granules, including enlarged cells protruding into outflow tract vessels (Fig. 3B,C,F,G,J,K, red arrowheads). There was no evidence of collapse of inter-trabecular spaces or physical obstruction of the TM or collector channels by pigment (Fig. 3B and C). Ultrastructurally, pigment granules induced activation of lysosomes and phagosomes *ex vivo* and *in vitro* (Fig. 3K and L, blue arrowheads). Many pigment granules appeared to be ingested at different stages of hydrolysis by secondary lysosomes (Fig. 3K and L). A swollen and distended endoplasmic reticulum was frequently observed in these eyes (Fig. 3J and L, yellow arrowheads).

### Decreased phagocytosis and disruption of the cytoskeleton

Reorganisation of the cytoskeleton^41^ and reduced phagocytosis^42,43^, features related to regulation of outflow^44^, were the most notable observations in eyes exposed to pigment.

**Figure 4:**
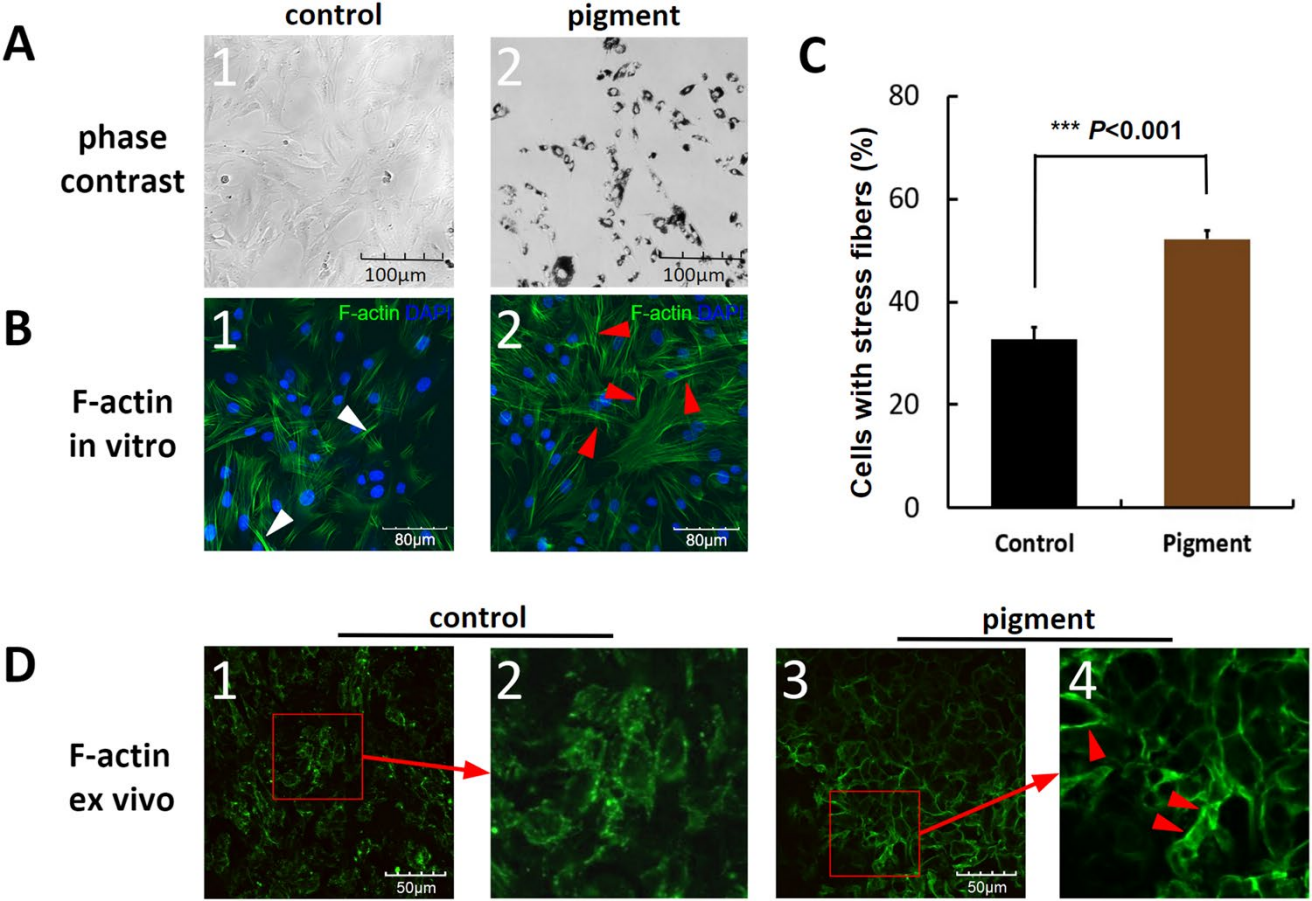
Cytoskeletal changes in the trabecular meshwork induced by pigment. Primary TM cells (**A1**) did not show morphological changes when exposed to pigment granules (**A2**). Normal F-actin cytoskeleton (white arrowheads, **B1**). Pigment-induced actin stress fibres with long, thick bundles (red arrowheads, **B2**). Actin stress fibers increased from 32.64 ± 2.37% in controls to 52.16 ± 1.69% in the pigment group (p < 0.001). The F-actin cytoskeleton of flat-mounted normal TM tissue with weak segmental structures (**D1,D2**), in contrast with thick continuous stress fibers in the pigment group (**D3,D4**). TM, trabecular meshwork.

Primary TM cells normally have a flat and elongated body with drawn-out tips and then assume a spindle shape as confluence increases (Fig. 4A1). This was not significantly affected by treatment with pigment (Fig. 4A2). We used phalloidin to label the F-actin cytoskeleton of TM cells. Normal F-actin fibers presented as well-organized, fine, feather-like microfilaments (Fig. 4B1, white arrowheads), whereas the pigment caused polymerization of F-actin fibers into long thick bundles (Fig. 4B2, red arrowheads). Quantitative analysis showed a significantly higher percentage of TM cells containing actin stress fibers in the pigment-treated group than in the control group (32.64 ± 2.37% vs 52.16 ± 1.69%, p < 0.001; Fig. 4C).

Consistent with the *in vitro* findings, the F-actin microfilaments in the normal flat-mounted TM tissue samples showed weak, spot-like, or segmental staining (Fig. 4D1 and D2) that contrasted with the thick, bundle-like, continuous stress fibers in the pigment group (Fig. 4D3 and D4).

**Figure 5:**
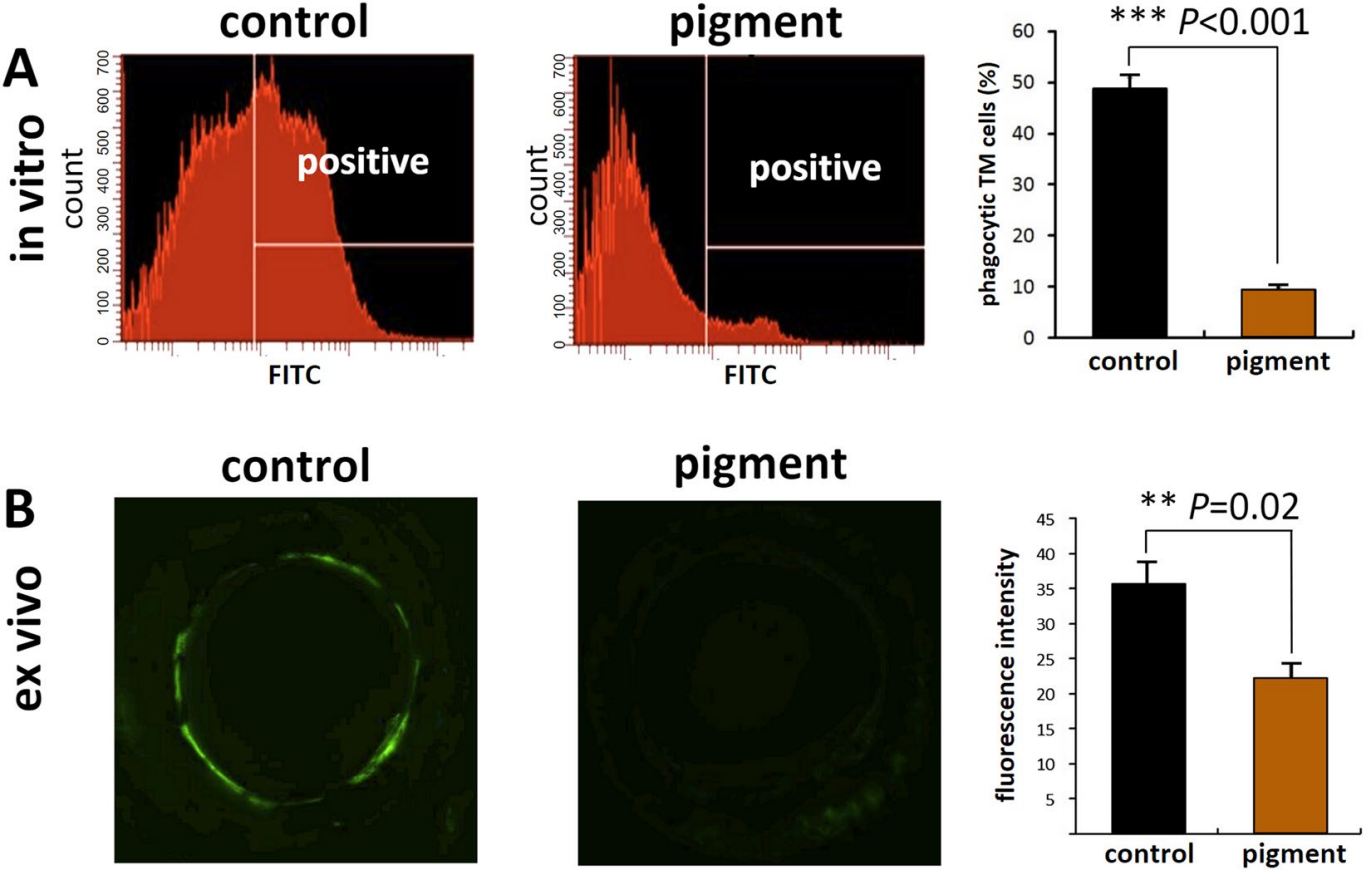
Phagocytosis in the trabecular mess network. *In vitro* TM phagocytosis was quantified by flow cytometry. Normal primary TM cells phagocytose fluorescent microspheres, but pigment dispersion reduced uptake by 5.17-fold (48.73 ± 2.17% vs 9.43 ± 4.2%, p < 0.001 **[A]**). *Ex vivo* TM phagocytosis was quantified in a similar fashion but by measuring fluorescence of inverted anterior segments instead (**B**) inverted culture dish with a direct view of the entire fluorescent meshwork^17,22^). Compared with the controls, fluorescence was significantly lower (3.4 × 10^7^ ± 4.5 × 10^6^ vs 2.2 × 10^7^ ± 2.1 × 10^6^, p = 0.020; **p < 0.01; ***p < 0.001). TM, trabecular meshwork.

*In vitro* phagocytosis was measured by flow cytometry. Normal primary TM cells readily phagocytosed carboxylate-modified green-yellow microspheres. In contrast, cells exposed to pigment showed a 5.17-fold decreased uptake (controls, 48.7 ± 2.17%; eyes with pigment dispersion, 9.4 ± 4.2%; p < 0.001; Fig. 5A). We also developed a semiquantitative method to measure phagocytosis in the TM *ex vivo*. Carboxylate-modified microspheres were perfused into the anterior chambers to be phagocytosed by TM cells *in situ*. Fluorescent intensity could be observed using an epifluorescence-equipped dissecting microscope only after uptake. The raw TM fluorescent intensity was significantly higher in the control group than in the pigment group (3.4 × 10^7^ ± 4.5 × 10^6^ vs 2.2 × 10^7^ ± 2.1 × 10^6^; p = 0.020) (Fig. 5B).

**Figure 6:**
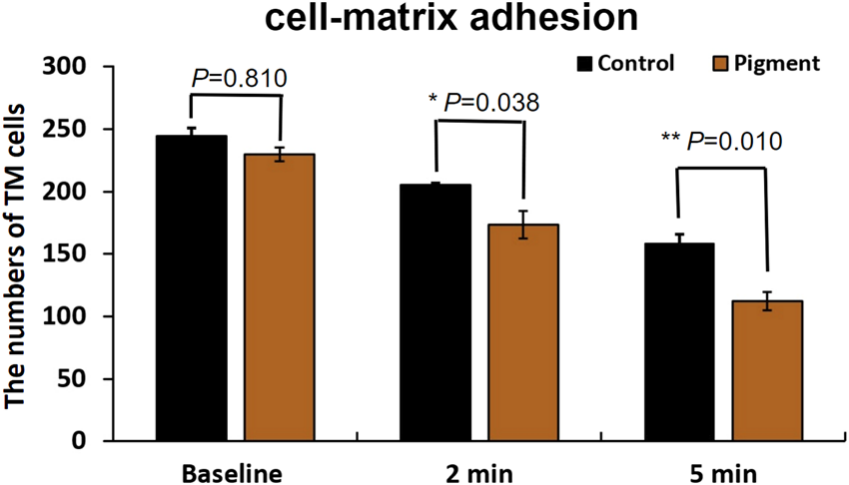
Cell matrix adhesion. In the pigment group, there were significantly fewer cells in the trabecular meshwork at 2 min (173.33 ± 10.81 vs 205.00 ± 1.53, p = 0.038) and 5 min (112.33 ± 11.30 vs 158.67 ± 6.94), p = 0.010), compared to the control (left).

We also quantified the cell-matrix adhesion as previously described^45^. Confluent TM monolayers exposed to pigment or vehicle treatment were subjected to trypsinization to measure cell-matrix adhesion. The numbers of TM cells per visual field were not significantly different between the pigment group and the control group before trypsinization (230.00 ± 5.51 vs 244.33 ± 6.39, p = 0.810). After trypsinization, more TM cells in the pigment group started to contract and detach. Fewer TM cells remained in the pigment group than in the control group at 2 min (173.33 ± 10.81 vs 205.00 ± 1.5; p = 0.038) and 5 min (112.33 ± 11.30 vs 158.67 ± 6.94; p = 0.010; Fig. 6).

**Figure 7:**
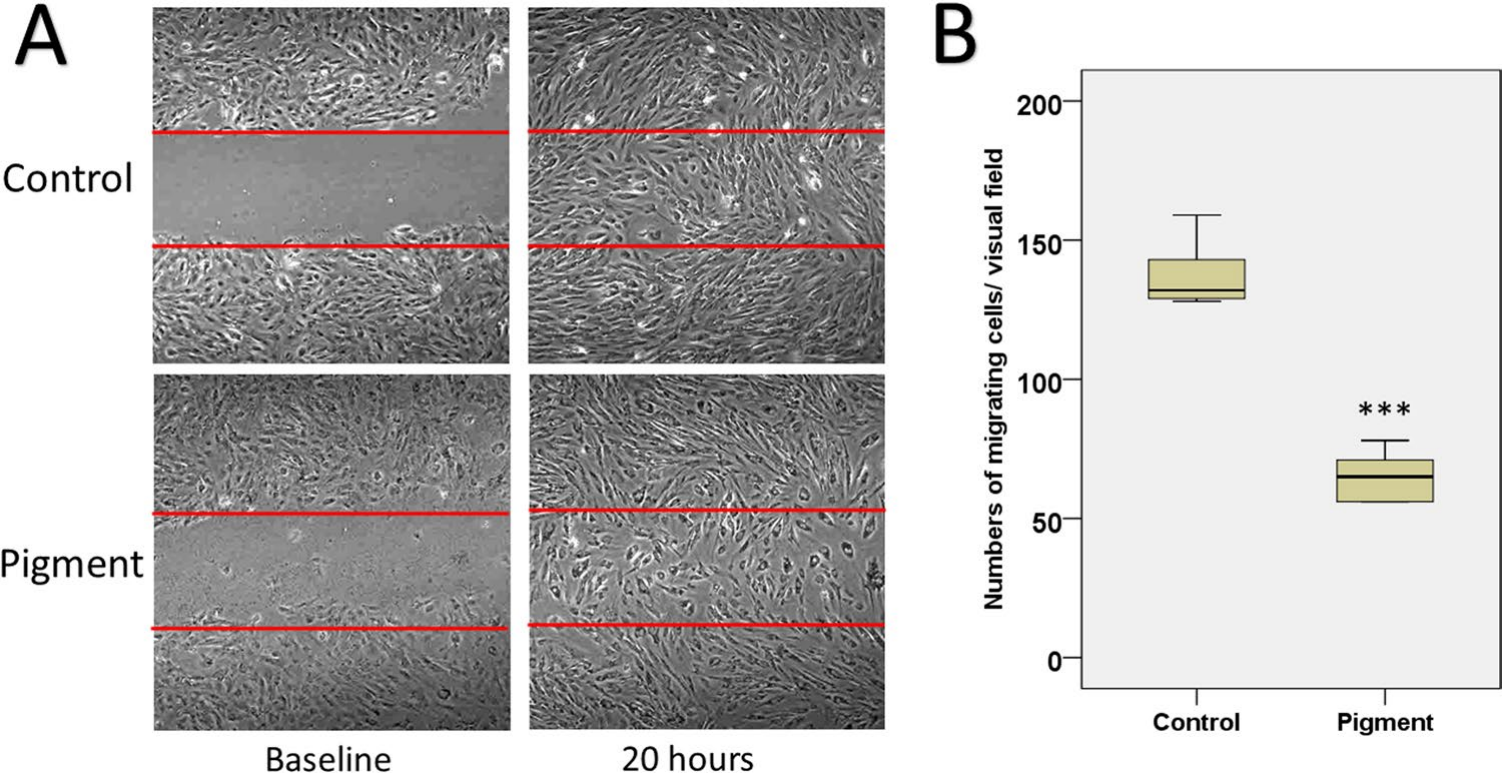
Cell migration. TM migration was assessed by a scratch assay **(A)**. Compared to the control, the pigment treatment significantly reduced the numbers of migrating TM cells by 47.53% in a 20-hours observation (137.17 ± 4.88 versus 65.20 ± 4.28, P < 0.001) **(B)**.

Cell migration was assessed by a scratch assay. After 20 hours of exposure to pigment granules, the migration was reduced by 47.5% (137.17 ± 4.88 versus 65.20 ± 4.28 in the control, P < 0.001) **(**Fig. 7**)**.

### Pathways of cell movement, phagocytosis, and aqueous outflow

**Figure 8:**
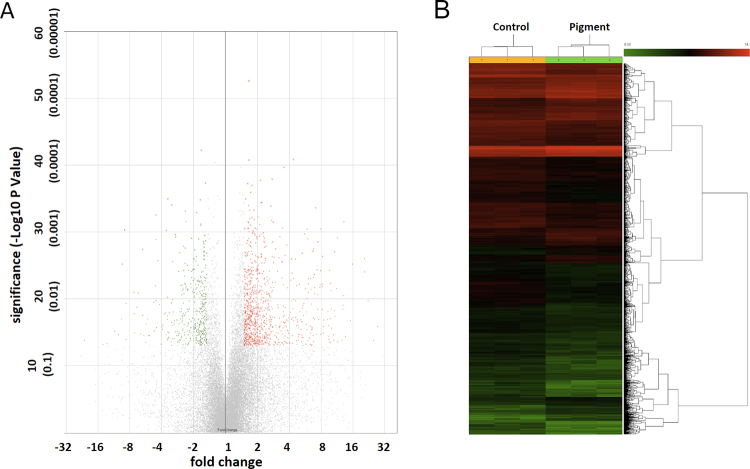
Differential gene expression by pigment treatment. Three TM samples from eyes that had a confirmed intraocular pressure elevation phenotype were compared with controls using the Affymetrix Gene 3′ IVT Microarray. A total of 24,123 porcine genes were hybridized, of which 691 were upregulated (red dots in volcano plot and red lines in heatmap) and 332 were downregulated (green dots in volcano plot and green lines in heatmap) by more than 1.5-fold with a p-value of ≤ 0.05. TM, trabecular meshwork.

Three *ex vivo* TM samples from each group were submitted for analysis using a gene expression microarray. A total of 24,123 porcine genes were analyzed, of which 691 were upregulated (red dots in volcano plot Fig. 8A and red lines in heatmap Fig. 8B) and 332 were downregulated by more than 1.5-fold (green dots in volcano plot in Fig. 8A and green lines in heatmap Fig. 8B and Supplemental Table 1, p < 0.05).

**Figure 9:**
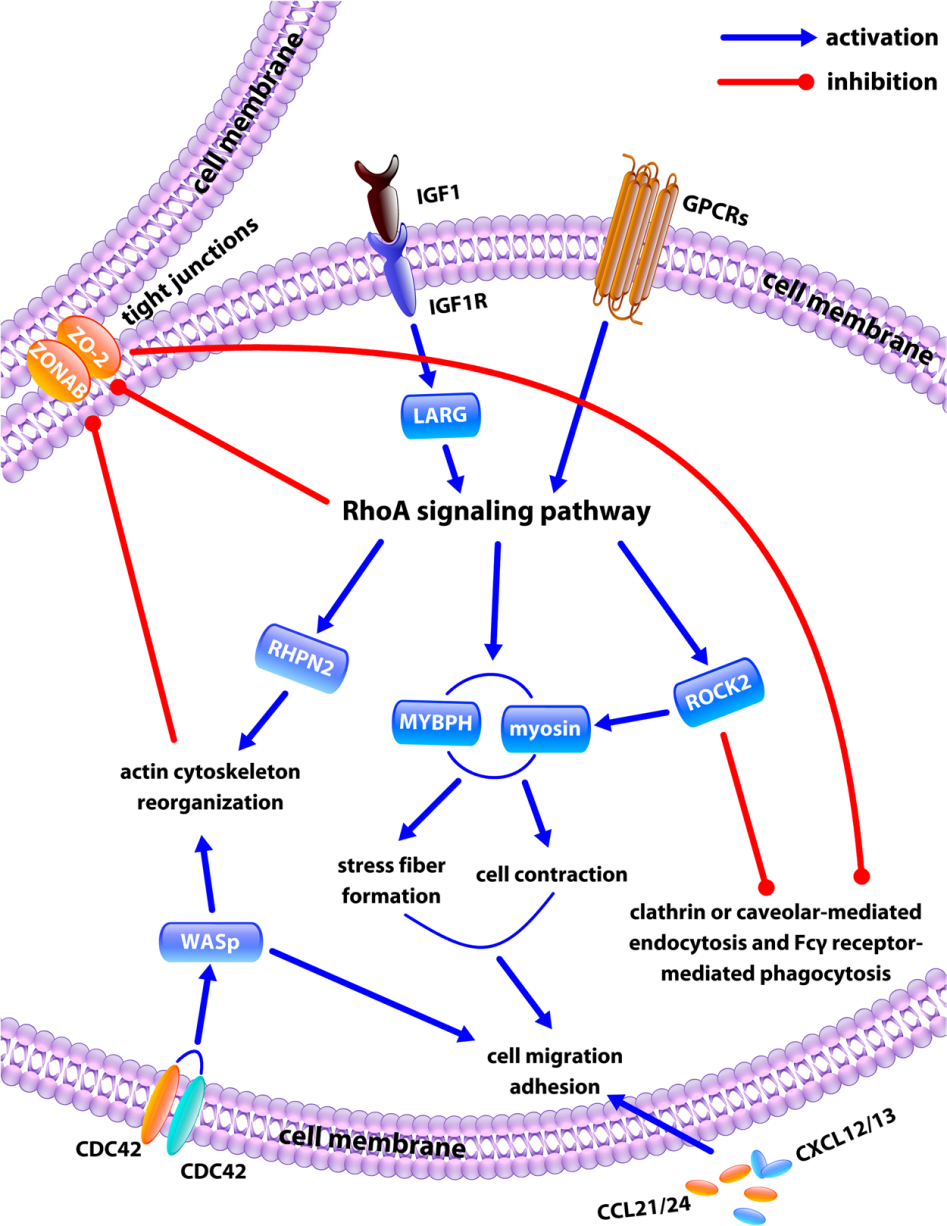
Model based on Ingenuity Pathway Analysis of trabecular meshwork expression exposed to pigment. RhoA signal is initialized by a complex consisting of the insulin growth factor (IGF), the type 1 insulin-like growth factor receptor (IGF-IR), and the lysophosphatidic acid receptor (LPAR).

After excluding 239 porcine genes with unclear biological functions, 784 genes (Supplemental Table 2**)** were mapped in our pathway analysis to 16 distinct signaling pathways. These genes were related to (1) cellular movement (cell adhesion, diapedesis, and migration), (2) endocytosis and phagocytosis, (3) aqueous outflow facility, (4) oxidative stress and endoplasmic reticulum stress, and (5) remodeling of the extracellular matrix of the TM **(**Fig. 9**)**. Key genes and signal pathways are summarized in Table 1 while their upstream regulators are listed in Supplemental Table 3. Genes involved in cell movement were significantly upregulated (Supplementary Figure 1).

**Table 1:** Expression changes of key genes and their pathways after exposure to pigment as indicated by pathway analysis.

Gene symbol	Entrez gene name	Fold change	*P*-value (ANOVA)	Signal pathways	Biological functions
LPAR3	lysophosphatidic acid receptor 3	6.02	0.042	eNOS signal pathway, VEGF signal pathway, RhoA signal pathway and Gα12–13 signal pathway	Outflow regulation
PGF	placental growth factor	2.49	0.00164	Ephrin receptor signal pathway, VEGF signal pathway, eNOS signal pathway, clathrin-mediated endocytosis signal pathway, IL-8 signal pathway, and hepatic fibrosis signal pathway	Outflow regulation; extracellular matrix remodeling; cell adhesion and migration
PIK3R2	phosphoinositide-3-kinase regulatory subunit 2	2.13	0.0389	IL-8 signal pathway, VEGF signal pathway, eNOS signal pathway,NRF2-mediated oxidative stress response signal pathway, Gα12–13 signal pathway, clathrin-mediated endocytosis signal pathway, Fcγ receptor-mediated phagocytosis signal pathway, JAK-STAT signal pathway	Outflow regulation; cell migration; cell phagocytosis; oxidative stress and endoplasmic reticulum stress
ALB	albumin	2.11	0.0299	Caveolar-mediated endocytosis signal pathway, and clathrin-mediated endocytosis signal pathway	Cell endocytosis
WAS	Wiskott-Aldrich syndrome	2.1	0.0439	Ephrin receptor signal pathway, Fcγ receptor-mediated phagocytosis signal pathway	Cell adhesion and migration
CDH5	cadherin 5	2.05	0.0119	Agranulocyte adhesion and diapedesis signal pathway, and Gα12–13 signal pathway	Cell adhesion; outflow regulation
KDR	kinase insert domain receptor	2.02	0.000598	eNOS signal pathway, and VEGF signal pathway	Outflow regulation
FLT1	fms related tyrosine kinase 1	1.98	0.0319	eNOS signal pathway, and VEGF signal pathway	Outflow regulation
ITGB8	integrin subunit beta 8	1.96	0.0121	Caveolar-mediated endocytosis signal pathway, and clathrin-mediated endocytosis signal pathway	Cell endocytosis
CXCL12	C-X-C motif chemokine ligand 12	1.9	0.0264	Agranulocyte adhesion and diapedesis signal pathway, and ephrin receptor signal pathway	Cell adhesion and migration
IGF1-R	insulin like growth factor 1 receptor	1.8	0.0165	PETN signal pathway, RhoA signal pathway, hepatic fibrosis signal pathway	Extracellular matrix remodeling; cell adhesion; outflow regulation
PIK3C2B	phosphatidylinositol-4-phosphate 3-kinase catalytic subunit type 2 beta	1.64	0.0118	IL-8 signal pathway, VEGF signal pathway, eNOS signal pathway,NRF2-mediated oxidative stress response signal pathway, Gα12–13 signal pathway, clathrin-mediated endocytosis signal pathway, JAK-STAT signal pathway	Outflow regulation; cell migration; cell endocytosis; oxidative stress and endoplasmic reticulum stress
LPAR6	lysophosphatidic acid receptor 6	1.56	0.0456	eNOS signal pathway, VEGF signal pathway, RhoA signal pathway, and Gα12-13 signal pathway	Outflow regulation
IGF-1	insulin like growth factor 1	1.54	0.0452	Clathrin-mediated endocytosis signal pathway, RhoA signal pathway, hepatic fibrosis signal pathway	Extracellular matrix remodeling; cell endocytosis; outflow regulation
FYN	FYN proto-oncogene, Src family tyrosine kinase	−1.51	0.0454	Ephrin receptor signal pathway, Fcγ receptor-mediated phagocytosis signal pathway, caveolar-mediated endocytosis signal pathway	Cell adhesion and migration; cell phagocytosis; cell endocytosis
ROCK2	Rho associated coiled-coil containing protein kinase 2	−1.56	0.0116	Ephrin receptor signal pathway, RhoA signal pathway, VEGF signal pathway, Gα12-13 signal pathway, IL-8 signal pathway	Cell migration; cell adhesion; outflow regulation
CDH3	cadherin 3	−1.56	0.0114	Gα12-13 signal pathway	Outflow regulation
PKC-Z	protein kinase C zeta	−1.75	0.0488	IL-8 signal pathway, PETN signal pathway, eNOS signal pathway, NRF2 mediated oxidative stress response signal pathway, Fcγ receptor-mediated phagocytosis signal pathway	Cell migration; cell adhesion; cell phagocytosis; oxidative stress and endoplasmic reticulum stress; outflow regulation
ITGA6	integrin subunit alpha 6	−1.77	0.0458	Agranulocyte adhesion and diapedesis signal pathway, and caveolar-mediated endocytosis signal pathway	Cell adhesion; cell endocytosis
ITGA2	integrin subunit alpha 2	−1.8	0.00624	Ephrin receptor signal pathway, caveolar-mediated endocytosis signal pathway	Cell adhesion and migration
VEGFA	vascular endothelial growth factor A	−2.45	0.0244	VEGF signal pathway, eNOS signal pathway, clathrin-mediated endocytosis signal pathway, ephrin receptor signal pathway, IL-8 signal pathway, hepatic fibrosis signal pathway	Extracellular matrix remodeling; cell adhesion and migration; cell endocytosis; outflow regulation
DNAJC3	DnaJ heat shock protein family (Hsp40) member C3	−2.56	0.00741	Unfolded protein response signal pathway, NRF2-mediated oxidative stress response signal pathway	Oxidative stress and endoplasmic reticulum stress; oxidative stress and endoplasmic reticulum stress
DNAJB9	DnaJ heat shock protein family (Hsp40) member B9	−3.12	0.0157	Unfolded protein response signal pathway, NRF2-mediated oxidative stress response signal pathway	Oxidative stress and endoplasmic reticulum stress; oxidative stress and endoplasmic reticulum stress
CLDN2	claudin 2	−11.08	0.0305	Agranulocyte adhesion and diapedesis signal pathway	Cell adhesion;

## Discussion

In this study, we developed a hypertensive pigment dispersion model in *ex vivo* pig eyes to study the function and signal pathway changes of the TM. Ocular hypertension in pigment dispersion syndrome can lead to pigmentary glaucoma (PG), a common and often risky type of secondary glaucoma in humans. To the best of our knowledge, this is the first *ex vivo* system to do so in any species. Analogous to a freeze-thaw decellularization technique we developed recently^46^, we used a freeze-thaw technique to produce pigment granules derived from the iris while preserving epitopes and avoiding use of chemicals. In PG, iridozonular contact and pupillary movement^47^ cause loss of cells within the iris stroma and the pigmented iris epithelium^48–50^. Our pigment granules were similar in size to those observed in human PG^9^. These induced a hypertensive IOP phenotype without physical obstruction of the intertrabecular spaces, similar to that occurring in human patients with PG^46^. Our results suggest that ocular hypertension in PG is associated with the formation of actin stress fibers, reduced phagocytosis, and cell-matrix adhesion.

We isolated primary porcine TM cells for *in vitro* experiments. These TM cells displayed the typical TM markers^51^ seen in human TM cells and readily phagocytosed particulate matter. The IOP started to increase in eyes with continuous pigment dispersion after 48 hours and remained at a similar level thereafter. These eyes also displayed a larger range of IOP values than the normal controls, indicating a variety of individual IOP responses, an observation that correlates with the IOP in pigment dispersion syndrome and PG in human patients^28^. An elevation of IOP to 10 mmHg above baseline is comparable with the clinical presentation of PG^3,4^.

The ultrastructure and histological findings were consistent with those in studies of human eyes^9,27,52,53^. Most importantly, there was no physical obstruction to outflow, rather a relatively modest deposition of pigment within the TM and its cells. In the anterior segments with pigment diffusion, phagocytosis and breakdown of ingested pigment granules continued in the cells of the TM. The TM cytoskeleton displayed stress fibers with polymerization of F-actin microfilaments. In contrast, with the formation of cross-linked actin networks that occurs in other forms of glaucoma^54^, for instance, steroid-induced glaucoma^55^ and primary open-angle glaucoma^56^, the stress fibers in this model were long, thick, bundle-like microfilaments. Human TM cells could form cross-linked actin networks within 7 days in steroid-induced ocular hypertension^54,55^. Acute disruption of F-actin was reported in bovine TM cells that underwent phagocytic challenge induced by latex microspheres^57^. Cytoskeletal changes in the TM that directly affect its stiffness and outflow facility^58^ have recently been targeted with newer glaucoma medications in the form of ROCK inhibitors^59^ and NO donors^60^ that relax the TM. However, dexamethasone^61^, TGF-β2^62^, and senescence^63^ result in increased stiffness of the extracellular matrix and decreased outflow.

The TM not only regulates outflow but also prevents debris from entering the outflow system by phagocytosing it and presenting it to the immune system^64,65^. Genes for cell motility were significantly upregulated, possibly reflecting migration of TM cells into Schlemm’s canal, as has been reported in human PG^9,27^. We observed a decline in cell-matrix adhesions and an altered cytoskeleton, as reported in another study^66^. It is possible that a limited amount of pigment may cause no harm, but when a threshold is exceeded, the phagocytic capacity is overwhelmed, as evidenced by reduced uptake of fluorescent spheres. TM cells may then migrate from the extracellular matrix of the TM and potentially occlude the intertrabecular space or the downstream outflow tract. This may explain why the pathology of TM in PG is so different from that in pigment dispersion syndrome^28^. Gottanka *et al*.^9^ found that the Schlemm’s canal in human PG specimens was partially (25%–65%) obstructed by loosely arranged, distended juxtacanalicular TM cells.

The gene expression changes in this model suggest a central role for RhoA and its activators (IGF/IGF1R/LPAR) in the molecular pathogenesis of PG with altered actin cytoskeleton and cell motility^67^. According to the pathway modeling, RhoA signaling, the central pathway that regulates the actin cytoskeleton of the TM, is initiated by exposure to pigment via a complex consisting of insulin growth factor (IGF), the type 1 insulin-like growth factor receptor (IGF-IR), and the lysophosphatidic acid receptor (LPAR) in the cell membrane. This is different from the RhoA activation induced by transforming growth factor (TGF)-β and its receptor in primary open-angle glaucoma^68^ and steroid glaucoma^69^. In addition to the inhibition of tight junctions by RhoA, upregulation of the rhophilin Rho GTPase binding protein would also promote reorganizing the TM actin cytoskeleton. This would negatively affect the tight junction protein 2/zonula occludens-associated nucleic acid binding protein complex (TJP2/ZONAB), and inhibit TM tight junction and clathrin, caveolae, or Fcγ receptor-mediated endocytosis and phagocytosis. The pathway analysis indicated that activation of RhoA signaling also enhances the myosin/MYBPH-mediated actin polarization, formation of stress fibers, and contractility of the TM. A change in TM motility could also result from upregulation of a set of chemokine ligands (CCL21/CCL24 and CXCL12/CXCL13) in the cell membrane and Wiskott-Aldrich syndrome protein in the cytoplasm. Activation of the RhoA pathway is seen in primary open-angle glaucoma^68^ and in steroid-induced glaucoma^69^. In contrast, with TGF-β-mediated RhoA activation in primary open-angle glaucoma^68^ and steroid-induced glaucoma^69^, this process seems initiated by IGF1. IGF1 has been implicated in ocular neovascularisation^70^, and it is tempting to speculate about a role in the uveitis-hyphema-glaucoma syndrome^71^. In that syndrome, intraocular lens implants cause chronic pigment release, atrophy of the iris, and hyphema. In our study, transcription of LPAR3 and LPAR6 was upregulated 6.02 and 1.56 times, respectively, suggesting LPA/LPAR signaling. LPARs bind to Gα12/13 and activate Rho-Kinase/ROCK^72–74^. Inhibition of LPA/LPAR signaling by the autotaxin inhibitor S32826 could reduce IOP in a rabbit glaucoma model^75^.

The tight junctions in the TM are formed by interaction between actin fibers and tight junction proteins. Junctional tightness and distribution influence aqueous outflow facility and cell adhesion^76^. The downregulation of CDLN2 may be related to increased cell migration^77^. Activation of RhoA was reported to disrupt TM tight junctions by modulating actin stress fibres^78,79^. Downregulation of CLDN2 and ZO-2 may also point to increased migration and have been seen after corticosteroids^79^. Normal tight junctions help maintain cell polarity for specialized surface functions, like receptor-mediated phagocytosis and endocytosis. The expression microarray and pathway analysis suggested that endocytosis pathways mediated by clathrin or caveolin and the Fcγ receptor-mediated phagocytosis pathway were downregulated.

The advantage of the pig eye *ex vivo* model is that eyes are similar in size to human eyes, are readily available from local abattoirs^16,17,22,23,26,46^, can be cultured *ex vivo*^17^, the TM can be observed through the bottom of the culture dish^17^, agents (vectors, pigment, drugs) can be applied continuously, techniques to measure regionally discrete outflow exist^16,23^, ample TM material can be obtained from a single animal (helpful in gene expression microarrays), and any potential immune response to injection of material from other animals^14^ is eliminated *ex vivo*. However, this model also has several limitations: compared with human PG, the IOP elevation seen here was relatively acute and took only 2 days from the time of pigment exposure to onset of ocular hypertension. Yet, although pigment showers can cause IOP spikes, consistently high IOP in human patients can take years to develop^80,81^. *In vivo*, clearing of debris by macrophages, in addition to the phagocytosis by TM cells, is more effective^64,65^. While the pigment granules used here look like those seen in human PG and are of a comparable mixed epithelial and stromal origin, it is not clear what component is primarily responsible for the IOP elevation. Just because the dark color of melanin is visually striking does not mean it is causatively linked. To determine what cellular fragments, organelles or proteins might contribute the most, careful fractionation experiments have to be performed. Finally, the transcriptional changes in the LPA, IGF and ROCK pathways still need to be verified at a protein level and pathway activators or inhibitors need to be tested whether they can prevent or induce the hypertensive phenotype.

In summary, this model of pigment dispersion manifests a hypertensive IOP phenotype and has histological and ultrastructural characteristics similar to those in human PG. Pathway analysis revealed that activation of the RhoA pathway played a central role in the actin cytoskeleton, formation of tights junction, phagocytosis, and cell motility in the TM, each of which might provide new targets.

## Methods

### Generation of pigment

This study was designated as having exempt status by the Institutional Review Board of the University of Pittsburgh under section 45 CFR 46.101(b)(1) of the Code of Federal Regulations. Ten fresh pig eyes were obtained from a local abattoir (Thoma Meat Market, Saxonburg, PA) within 2 h of sacrifice, decontaminated for 2 min with 5% povidone-iodine ophthalmic solution (Betadine 5%; Alcon, Fort Worth, TX, USA), and hemisected. After removal of the posterior segment, lens, and ciliary body, the anterior segments were saved in plain Dulbecco’s Modified Eagle Medium for mounting. The irises were collected to produce pigment granules with two freeze-thaw cycles. Briefly, 10 irises were placed in 15 ml of phosphate-buffered saline (PBS), frozen at −80 °C for 2 h, and then thawed at room temperature. After two cycles of freeze-thaw, the tissue became fragile, and shed pigment when pipetted up and down 100 times with a 3-ml Pasteur pipette. The pigment granules were filtered through a 70-μm cell strainer (cat#431751, Corning Incorporated, Durham, NC, USA). After spinning at 3000 rpm for 15 min, the supernatant was discarded, and the pigment granules were resuspended in 15 ml of PBS and centrifuged again. Finally, the pellet was resuspended in 4 ml of PBS and stored at 4 °C as a stock suspension.

The concentration of pigment granules was measured by hemocytometer counts (cat#1490, Hausser Scientific, Horsham, PA)^14^. The stock suspension was diluted 1000-fold and visualized by a phase-contrast microscope at 600x magnification (Eclipse TE200-E, Nikon Instruments Inc., Melville, NY, USA). Particle size was calculated as the average of 100 particles.

### Primary TM cell culture

A glaucoma surgeon experienced in microincisional glaucoma surgery^24–26,82–86^ carefully excised the TM from each freshly enucleated pig eye under an ophthalmic operating microscope (Stativ S4, Carl Zeiss, Oberkochen, Germany). Each TM was sectioned into pieces measuring 0.5 × 0.5 mm, and maintained in TM medium (OptiMEM; 31985–070, Gibco, Life Technologies, Grand Island, NY, USA) supplemented with 5% fetal bovine serum and 1% antibiotics (15240062, Thermo Fisher Scientific, Waltham, MA, USA) in a humidified CO_2_ incubator at 37 °C. Primary TM cells migrated out of the tissues and formed clones on day 5. After 100% confluence was reached, the cells were trypsinized for 5 min, centrifuged at 1000 rpm for 3 min, and replated at a ratio of 1:3. To reduce the chances of differentiation, only the first four passages of cells were used^87^.

Primary TM cells were authenticated both by immunostaining with TM-specific antibodies and phagocytosis testing. Briefly, the cells were fixed in 4% paraformaldehyde for 1 h, washed with PBS three times, and incubated with the following primary antibodies at 4 °C overnight: goat polyclonal MGP antibody (1:100 dilution in PBS, sc-32820, Santa Cruz Biotechnology, Dallas, TX, USA), rabbit polyclonal anti-alpha-SMA; 1:100, ab5694, Abcam, Cambridge, MA, USA), and AQP1 antibodies (1:100, Sc-20810, Santa Cruz Biotechnology). After three rinses in PBS, donkey-anti-goat Alexa Fluor 647 (1: 1000, ab150131, Abcam) and goat anti-rabbit IgG secondary antibodies were added for 45 min at room temperature. Cell nuclei were counterstained with DAPI (D1306, Thermo Fisher Scientific). Pictures were taken using an upright laser scanning confocal microscope at 400x magnification (BX61, Olympus, Tokyo, Japan).

To further validate the primary TM cells, we also performed a phagocytosis activity assay and steroid induction of myocilin (MYOC). For the phagocytosis test, cells at 80% confluency were incubated for one hour with carboxylate-modified yellow-green fluorescent microspheres (0.5 micron diameter, cat# F8813, Thermo Fisher, Waltham, MA) at a final concentration of 5 × 10^8^ microspheres/ml. This was followed by three rinses with prewarmed PBS. To visualize TM phagocytosis, pictures were taken with a phase contrast fluorescence microscope at 200x magnification. Cells were then trypsinized, and resuspended in 500 µl PBS for flow cytometry. The percentage of TM cells that had ingested fluorescent microspheres was determined. For the steroid-induced myocilin assay, the cells at 80% confluency were treated with Dex at 500 nM or PBS vehicle for 7 days, then were lysed with TriZol reagent (15596026, Invitrogen, Thermo Fisher, Waltham, MA) for RNA extraction, reverse transcription (4368814, Thermo Fisher Scientific, Waltham, MA) and qPCR (4376600, Thermo Fisher Scientific, Waltham, MA) in accordance with the manufacturer’s instructions. The primers used in the experiment were as follows: MYOC sense: GGTCATTCCGGCAGTGAAGAA, MYOC antisense: ACGCCGTACTTGCCAGTGATT; GAPDH sense: CCCCACCACACTGAATCTCC, GAPDH antisense: GGTACTTTATTGATGGTACATGACAAG.

### Cell viability assay

Primary TM cells were plated onto a six-well-plate at a density of 1 × 10^5^ cells per well and maintained in TM medium containing 1.67 × 10^7^ pigment granules per ml. TM medium without pigment served as a control. The medium was changed every 3 days for up to 10 days. The cells were stained with calcein-AM (0.3 µM, C1430, Thermo Fisher Scientific) and PI (1 µg/ml, P1304MP, Thermo Fisher Scientific) for 30 min, followed by trypsinization and resuspension in 500 µl of PBS for flow cytometry. Viable TM cells have intracellular esterase activity that can convert non-fluorescent calcein AM to green fluorescent calcein, but do not allow red fluorescent PI to pass through the intact cell membrane and bind to the cell nucleus^39^. Accordingly, calcein-labeled TM cells were counted as viable while PI-stained cells were assumed to be dead or apoptotic.

### Pigmentary glaucoma model

For the *ex vivo* perfusion model, anterior segments from fresh pig eyes were mounted and perfused with Dulbecco’s modified Eagle’s medium supplemented with 1% fetal bovine serum and 1% antibiotics at a rate of 3 µl/min, as described previously^17,46^. IOP was measured intracamerally by a pressure transducer (SP844; MEMSCAP, Skoppum, Norway) and recorded using a LabChart software system (ADInstruments, Colorado Springs, CO, USA)^17,46^. The transducers were calibrated using a transducer tester (Veri-Cal, Utah Medical Products, Midvale, UT, USA). Baseline IOP values were obtained after 72 h. Pigment granules diluted with perfusion medium to a concentration of 1.67 × 10^7^ particles/ml were perfused for 180 h. Normal medium without pigment granules served as a control. IOP values were recorded at 2-min intervals. Outflow facility which represents outflow resistance in the ocular drainage pathway was calculated by the classic Goldmann equation^40^. Outflow facility was the ratio of infusion rate to relevant IOP while the episcleral venous pressure was considered negligible.

For the *in vitro* studies, primary TM cells were treated with the pigment-containing medium and the control group was sham-treated. Briefly, 3 × 10^5^ primary TM cells were plated onto a 60-mm dish and maintained with OptiMEM supplemented with 5% fetal bovine serum and 1% antibiotics for 24 h. Pigment granules were then added to a final concentration of 1.67 × 10^7^ particles/ml. The medium was changed every 3 days. Normal TM medium without pigment served as a control.

### Histology and transmission electron microscopy

Anterior segments from perfusion cultures were fixed with 4% paraformaldehyde for 24 h, embedded in paraffin, cut into 5-μm sections, and stained with hematoxylin and eosin for histology^17^. The ultrastructure of *ex vivo* TM tissue and *in vitro* cell monolayers was evaluated by transmission electron microscopy. Preparation of samples for transmission electron microscopy followed a previous protocol with minor modification^88^. The samples were prefixed with 2.5% glutaraldehyde in 0.05 M cacodylate buffer for 24 h, washed in PBS three times, and then post-fixed with 1% osmium tetroxide solution overnight. After three rinses of PBS, the samples were dehydrated with an increasing ethanol series (30%, 50%, 70%, 90%, and 100%, for 45 min each), followed by embedding in Epon resin (Energy Beam Sciences, East Granby, CT, USA). The Epon resin was exchanged completely at hourly intervals for 3 h and blocks were cured for 2 days at 60 °C. Sections of 300 nm were obtained using a Reichert-Jung Ultracut 701701 Ultra Microtome and stained with 0.5% Toluidine Blue O Solution (S25613, Thermo Fisher Scientific). Ultra-thin sections of 65 nm were obtained and placed on grids. After staining with uranyl acetate and lead citrate, images were taken under an 80 kV JEOL transmission electron microscope (Peabody, MA) at various magnifications.

### Phagocytosis

For the *in vitro* phagocytosis assay, the primary TM cells exposed to pigment granules or sham treatment were incubated with fluorescent microspheres for 1 h, washed in PBS three times, trypsinized, resuspended, and then subjected to flow cytometry.

The *ex vivo* phagocytosis was quantified by measuring TM fluorescent intensity *in situ* after perfusion with carboxylate-modified yellow-green fluorescent microspheres for 24 h. Briefly, 0.5-μm carboxylate-modified yellow-green fluorescent microspheres at 5 × 10^8^/ml were added to the perfusion system for 24 h. The anterior chambers were washed three times with PBS. TM cells that had phagocytosed microspheres showed bright green fluorescence under a dissecting fluorescence microscope (SZX16, Olympus). Images were acquired at a pixel resolution of 680 × 510 and a 1/17-s exposure. The raw TM fluorescent intensity was measured using ImageJ version 1.50i software (National Institutes of Health, Bethesda, MD, USA) as described elsewhere^26,89^.

### TM cytoskeleton

F-actin was used to assess the cytoskeletal changes in the TM. *Ex vivo* and *in vitro* TM samples were fixed in 4% paraformaldehyde for 1 h and washed with PBS three times. The samples were incubated with Alexa Fluor 488 phalloidin (1:40 dilution, A12379, Thermo Fisher Scientific) for 30 min and counterstained with DAPI. Images were acquired using an upright laser scanning confocal microscope at 600-fold magnification (BX61, Olympus).

### TM motility

The cell-matrix adhesion was evaluated using a previously described protocol^45^. Confluent TM monolayers treated with pigment granules or sham were washed with PBS and dissociated with 0.25% trypsin. The changes in cell morphology and adhesion were monitored using a phase-contrast microscope at different trypsinization time intervals (0, 2, and 5 min). The ratio of the number of attached TM cells to total TM cells represented the cell-matrix adhesion. A scratch assay was conducted to quantify the *in vitro* TM cell migration. Briefly, 1 × 10^5^ primary TM cells were seeded into each well of a six-well-plate. At 100% confluent, a cell free scratch was gently made by a 10 ul pipette tip. The pigment dispersion group was exposed to 1.67 × 10^7^ particles/ml. Cell migration was monitored under a live cell imaging module. Six pictures were taken for each group at the baseline and the end of the study at 20 hours. The numbers of migrating cells were counted.

### Gene expression microarray and pathway analysis

Anterior segments (n = 3 each) from the pigment-treated and normal control groups were dissected after the IOP phenotypes were obtained. Cells from the TM were lysed with TRIzol (15596026, Invitrogen, Thermo Fisher Scientific) and sent to the Genomic Core Facility of the University of Pittsburgh. Amplification and hybridization were performed using the Affymetrix Porcine 3′IVT Array (900624, Affymetrix, Santa Clara, CA), which contains 23,937 probe sets to interrogate 23,256 transcripts in the pig and representing 20,201 *Sus scrofa* genes. The Affymetrix CEL files were extracted, normalized, and statistically analyzed by analysis of variance using the Transcriptome Analysis Console (TAC, version 3.1, Affymetrix). The differential gene expression profiles were characterized by a volcano plot and heatmap. The default filter criteria were fold change (linear) less than −1.5 or fold change (linear) above 1.5, and a p-value smaller than 0.05 (analysis of variance). Genes that matched these criteria were selected for bioinformatic pathway analysis using Ingenuity Pathway Analysis (Spring Release 2017, Qiagen, Hilden, Germany).

### Statistical analysis

The data are presented as the mean ± standard error to capture the uncertainty around the estimate of the mean measurement and to allow computation of the confidence interval. Differential gene expression was analyzed using TAC (Version 3.1, Affymetrix). Other quantitative data were processed by one-way analysis of variance using PASW version 18.0 (SPSS Inc., Chicago, IL, USA). A difference was considered statistically significant at p < 0.05.

### Data availability

All data generated or analyzed in this study are included in this published article and its Supplementary Information files.

## Electronic supplementary material

Supplementary Figure 1

Supplementary Information

Supplemental Table 1

Supplemental Table 2

Supplemental Table 3
