# Supplementary figures and images for: A porcine ex vivo model of pigmentary glaucoma
Source: Sci Rep. 2018 Apr 3;8:5468. doi: 10.1038/s41598-018-23861-x (PMC5882895; doi:10.1038/s41598-018-23861-x)

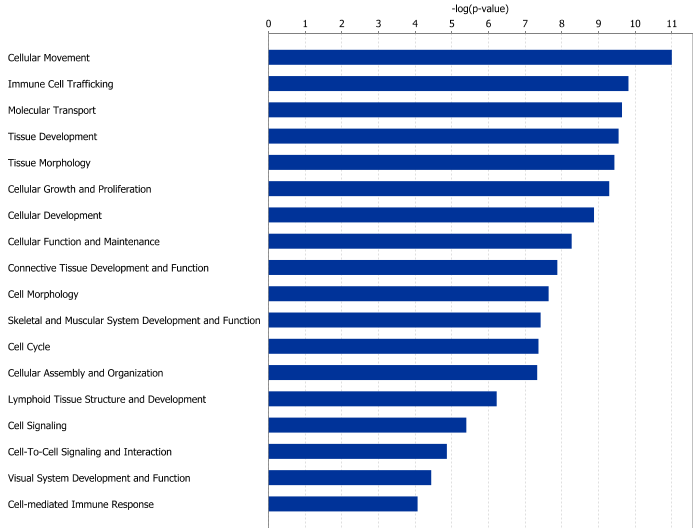

Supplement: Supplementary file 1 — Supplementary Figure 1 [file 41598_2018_23861_MOESM1_ESM.zip › Supplemental Figure 1 Cell motility function.tif]
