# Supplementary material for: A porcine ex vivo model of pigmentary glaucoma
Source: Sci Rep. 2018 Apr 3;8:5468. doi: 10.1038/s41598-018-23861-x (PMC5882895; doi:10.1038/s41598-018-23861-x)
Supplement: Supplementary file 2 — Supplementary Information [file 41598_2018_23861_MOESM2_ESM.doc]

# **A porcine ex vivo model of pigmentary glaucoma**

Yalong Dang^1^, Susannah Waxman^1^, Chao Wang^1, 2^, Ralitsa T. Loewen^1^, Ming Sun^3^ and Nils A. Loewen^1^

1: Department of Ophthalmology, School of Medicine, University of Pittsburgh, Pittsburgh, United States of America

2: Department of Ophthalmology, Xiangya Hospital, Central South University, Changsha, China

3: Department of Cell Biology, School of Medicine, University of Pittsburgh, Pittsburgh, United States of America

^#^Correspondence to loewen.nils@gmail.com

**Supplementary Figure 1.** Genes involved with cellular movement

**Supplemental Table 1.** Differential gene expression by pigment treatment

**Supplemental Table 2.** Gene mapped by Ingenuity Pathway Analysis

**Supplemental Table 3.** Upstream analysis
